# C-reactive protein expression in acute ischemic stroke blood clots: Implications for etiology

**DOI:** 10.1177/23969873251315636

**Published:** 2025-02-05

**Authors:** Wenyi Liu, Cansu Sahin, Nazan Güner Sak, Alice Giraud, Pierluca Messina, Franz Bozsak, Jean Darcourt, Federico Sacchetti, Anne-Christine Januel, Guillaume Bellanger, Jorge Pagola, Jesus Juega, Hirotoshi Imamura, Tsuyoshi Ohta, Laurent Spelle, Vanessa Chalumeau, Uros Mircic, Predrag Stanarčević, Ivan Vukašinović, Marc Ribo, Nobuyuki Sakai, Christophe Cognard, Karen Doyle

**Affiliations:** 1 Department of Physiology, University of Galway, Galway, Ireland; 2 CÚRAM Research Ireland Centre for Medical Devices, University of Galway, Galway, Ireland; 3 Sensome, Massy, France; 4 Department of Diagnostic and Therapeutic Neuroradiology, CHU de Toulouse, Toulouse, France; 5 Radiology Department, Ramsay Santé Clinique des Cèdres, Cornebarrieu, France; 6 Department of Neurology, University Hospital Vall d’Hebron, Barcelona, Spain; 7 Department of Neurosurgery, Kobe City Medical Centre General Hospital, Kobe, Japan; 8 Bicêtre Hospital, Department of Interventional Neuroradiology, Le Kremlin-Bicêtre, France; 9 Centre for Radiology and MRI, Clinic for Neurosurgery, University Clinical Centre of Serbia, Belgrade, Serbia; 10 Neurology Clinic, University Clinical Centre of Serbia, Belgrade, Serbia

**Keywords:** Stroke, inflammation, etiology, TOAST classification

## Abstract

**Introduction:**

C-reactive protein (CRP) is a prototypic inflammation marker, with elevated levels associated with an increased risk of cerebrovascular events. To determine whether CRP could be a useful biomarker of stroke etiology, we investigated CRP expression in acute ischemic stroke (AIS) clots from large-artery atherosclerosis (LAA), cardio-embolism (CE) and cryptogenic (Crypt) subtypes.

**Patients and methods:**

We analysed clot samples from AIS patients (LAA, CE, Crypt; *n* = 50 each), collected across five stroke centres in France, Serbia, Spain, and Japan between February 2021 and February 2024, as part of the prospective Clotbase International Registry of 460 patients who underwent mechanical thrombectomy. Clot components were assessed using Martius Scarlet Blue staining. CRP expression was examined using immunohistochemistry and its co-localisation with clot components was detected using immunofluorescence. Clinical parameters were compared across etiologies.

**Results:**

CRP expression varied significantly among clots. Most clots (65%) had minimal (⩽1%) CRP and 35% showed substantial (>1%) CRP. CE group had significantly more clots with substantial CRP than LAA and Crypt (48% vs 30% and 26%; *p* = 0.048). Clots with substantial CRP contained more fibrin (28.9%) than those with low CRP (20.6%; *p* = 0.005). Confocal microscopy showed CRP co-localised with fibrin and white blood cells (WBCs).

**Discussion and conclusion:**

Significantly more AIS clots of CE expressed substantial CRP compared to those of LAA and Crypt, suggesting CE strokes may be more strongly linked to inflammation. Clots with substantial CRP expression displayed significantly more fibrin compared to those with minimal CRP expression, suggesting a potential association between inflammation and fibrin-rich clots. Further study of the relationship between CRP, fibrin and WBCs in clots may improve our understanding of the processes of thrombo-inflammation.

## Graphical abstract

**Graphical abstract:**
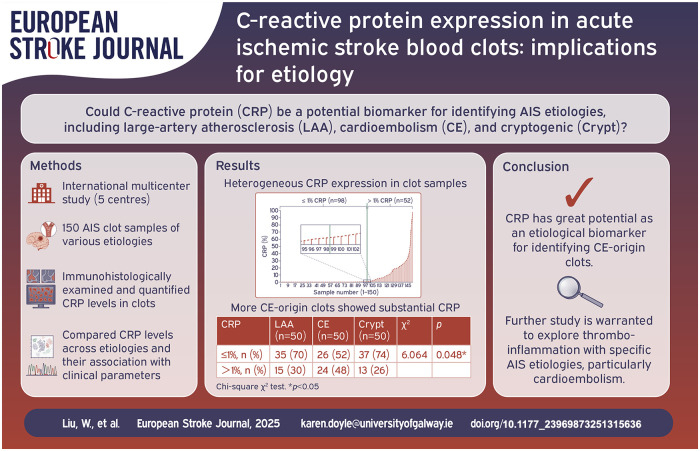

## Introduction

Stroke is a leading global health concern, ranking second in mortality and third in combined mortality and disability, with a serious impact on public health, quality of life, and economic burden.^1^ Among the various types of strokes, ischemic strokes are the most prevalent, accounting for approximately 87% of all stroke cases.^2^

CRP is a widely observed biomarker associated with inflammation, which is intricately linked to the pathogenesis of ischemic stroke.^3,4^ In AIS patients, CRP levels are significantly increased, and elevated plasma levels have been demonstrated as an independent predictor of unfavourable clinical outcomes.^5–7^ However, the involvement of CRP stratified by stroke etiology remains unclear.^8^ Numerous studies suggest that CRP could help identify the etiological subtypes of AIS, particularly in predicting CE strokes, as CRP levels are usually higher in AIS patients of CE-origin.^9–12^ Nevertheless, some other studies have not provided convincing evidence that CRP is associated with stroke etiologies,^13^ and there is still no consensus on the utility of CRP measurement in the clinical setting.^14^ Additionally, no evidence exists to show whether changes in CRP levels are causative or merely coincidental to more direct determinants of stroke etiology.^15^

Most existing studies focus on CRP levels in serum or plasma, leaving a gap in understanding its expression and function within thrombi. While these levels are well-established markers of systemic inflammation,^16^ examining CRP within thrombi provides a more direct approach to explore the role of inflammation in thrombogenesis. This approach could offer unique insights into the inflammatory processes involved in stroke, potentially bridging the divide between systemic inflammatory biomarkers and localized pathological events.

Although inflammation is increasingly recognised as a contributor to stroke, its relationship with specific stroke etiologies is not fully understood. Recent studies have emphasised the role of inflammation in atherosclerotic plaque rupture leading to LAA stroke.^17–19^ A recent meta-analysis reported that inflammation is strongly associated with LAA, Crypt, and small vessel occlusion strokes, but not CE stroke.^20^ This has led to the recruitment of patients with non-CE stroke in randomised controlled trials (RCTs) investigating anti-inflammatory therapies. However, inflammation has also been associated with the development of cardiac pathologies such as atrial fibrillation,^3,21^ which can lead to CE strokes. In support of this, several recent studies have reported higher WBCs levels in CE clots in comparison to other etiologies.^22–24^ Our current observations that high CRP levels are more common in CE clots also support the suggestion that inflammation may be an important contributor to the pathophysiology of CE strokes. Additional investigation into CRP could enhance our understanding of thrombo-inflammation in strokes of different etiologies. Further research is warranted to clarify the nuanced role of inflammation in CE and non-CE strokes to optimise patient selection in RCTs for future therapeutic interventions.

Our study represents the first investigation into CRP expression in thrombi, providing novel insights into its potential role in the pathophysiology of AIS. Specifically, we aim to investigate the association between CRP expression within thrombi of different AIS etiologies to further assess whether CRP could emerge as a possible etiological biomarker.

## Methods

### Study population

This study analysed clots from 150 patients diagnosed with AIS, sourced from the prospective Clotbase International Registry. Patients were cumulatively enrolled between February 2021 and February 2024. This multicenter study involved five hospitals across four countries: Purpan Hospital, France; Bicêtre Hospital, France; Vall d’Hebron Hospital, Spain; Clinical Centre of Serbia, Serbia; Kobe City Medical Centre General Hospital, Japan. Ethics approval for this study was granted by the University of Galway Research Ethics Committee (19-OCT-08). This study followed the General Data Protection Regulations, adhered to the principles of the Declaration of Helsinki, and complied with the Strengthening the Reporting of Observational Studies in Epidemiology (STROBE) guidelines.

### Inclusion and exclusion criteria

Enrollment included individuals with AIS who were 18 years or older and had furnished informed consent. Patients who had experienced an ischemic stroke due to a large vessel occlusion, undergone a mechanical thrombectomy (MT) procedure, and had a clot removed were included in this study. Additionally, patients were included regardless of their inflammatory or autoimmune conditions. No technical variables, including the administration of thrombolytics, the number of thrombectomy passes, the devices used, or the final modified Thrombolysis in Cerebral Infarction (mTICI) scores, were used as exclusion criteria.

### Baseline data collection

Experienced clinicians gathered the baseline data of the enrolled patients following a standardised data collection protocol. The collected data included information on age, sex, recombinant tissue plasminogen activator (rtPA) administration, etiological subtypes, risk factors, National Institute of Health Stroke Scale (NIHSS) scores at admission and discharge, modified Rankin Scale (mRS) scores at 90 days, final mTICI scores, and the number of required passes.

### TOAST classification

The TOAST classification system was used to define the stroke etiological subtypes. Stroke can be classified into five subtypes according to the TOAST classification system: large-artery atherosclerosis (LAA), cardio-embolism (CE), small-artery occlusion (lacune), stroke of other determined etiology and stroke of undetermined etiology (Crypt).^25^ Confirmed etiology was validated through a system that incorporated medical history and diagnostic tests, including brain imaging.

### Clot preservation, processing and cutting

Clot samples were collected by the MT technique. The clots were embedded in paraffin using standard processing protocol and stored at room temperature. They were cut into 3 μm sections using a microtome (Leica RM2125RTS).

### Martius scarlet blue (MSB) staining

Sections were baked at 60°C for 30 min, deparaffinized with xylene and hydrated through a graded alcohol series and distilled water. After immersion in Bouin’s solution at 56°C for 1 h, sections were stained with Iron ammonium-Celestine Blue and Mayer’s Hematoxylin for 10 min each. They were rinsed with 95% alcohol, stained with Martius Yellow and Crystal Scarlet, and differentiated in phosphotungstic acid. After that, they were stained with Methyl Blue, rinsed with aqueous acetic acid and dried under the fume hood. Finally, the sections were cleared in xylene and mounted with coverslips using DPX Mountant. This staining protocol was validated based on prior optimisation, confirming consistent and reproducible staining patterns for clot components.^26^ The staining specificity was further validated using both positive and negative tissue controls.

### Immunohistochemical (IHC) staining

IHC staining was performed using the Leica Bond III autostainer. The anti-CRP antibody (ab32412, Abcam) was diluted 1:400 with Bond Primary Antibody Diluent. Liver tissue sections served as controls. The Bond Polymer Refine Red Detection system was used for colour development. Antigen retrieval involved heat-induced epitope retrieval (HIER) with ethylenediaminetetraacetic acid (EDTA) for 20 min using BOND Epitope Retrieval Solution 2.

### Scanning and quantifying

The stained slides from MSB and IHC staining were scanned at 20× magnification using standard settings with the Olympus VS120 Digital Slide Scanner. After scanning, the images were used for quantification using Orbit Image Analysis Software (www.orbit.bio), as described previously.^27^

### Immunofluorescence (IF) staining

Sections were dehydrated at 60°C for 1 h, dewaxed in xylene, and then immersed in alcohol to remove residual xylene. Rehydration followed with immersions in 95%, 70% and 50% alcohol, and distilled water. Antigen retrieval involved heating sections in EDTA at 98°C for 20 min. After washing, they were incubated with 3% normal goat serum (NGS) for 1 h at 37°C, followed by primary antibody at 37°C for 1 h and overnight at 4°C. Primary antibodies used were as follows: anti-CRP (1:400, ab32412, Abcam), anti-Fibrinogen (1:1000, ab58207, Abcam), anti-CD66b (1:100, ab955, Abcam), anti-CD68 (1:2000, NB100-77808, Novus Biologicals), anti-CD3 (1:20, ab17143, Abcam), and anti-glycophorin A (1:100, ab212432, Abcam). The next day, they were incubated for 1 h with the secondary antibody: goat anti-rabbit IgG H&L (Alexa Fluor^®^ 488) (ab150077, Abcam) and goat anti-mouse IgG H&L (Alexa Fluor^®^ 594) (ab150116, Abcam). After washing, they were mounted with DAPI medium (ab104139, Abcam) and stored at 4°C for imaging with a confocal laser scanning microscope (FLUOVIEW FV3000, Olympus).

### Statistical analysis

Continuous variables from a normal distribution were represented using mean and standard deviation, while those from a non-normal distribution were represented using median and interquartile range (IQR). Categorical variables were represented using frequencies and percentages. Univariate comparisons used either Student’s *t*-test or the Mann–Whitney *U* test. Univariate multiple-group comparisons used one-way ANOVA or the Kruskal–Wallis test. Categorical variables were compared using the Chi-square test, Fisher’s exact test or Kruskal–Wallis test. All *p*-values were calculated using two-tailed tests, with significance level of 0.05. Analyses were performed using GraphPad Prism 10 and IBM SPSS Statistics 25.

## Results

### Patient characteristics

The clinical characteristics are detailed in Table 1. Male patients comprised 53.3%, slightly more than female patients (45.3%). The sex distribution differed significantly between etiological groups (*p* = 0.03), with a higher proportion of males in the LAA group (70%) compared to CE (46%) and Crypt (44%) groups. The median age was 71 years (IQR: 62–79). Age distribution differed significantly between etiological groups (*p* = 0.02), with CE patients being older (74; IQR: 68–82) than LAA (68; IQR: 62–78) and Crypt (70; IQR: 55–76). Atrial fibrillation, diabetes mellitus, and coronary artery disease were more common in CE (*p* < 0.001, *p* < 0.001 and *p* = 0.04, respectively). Fewer CE patients (22%) received rtPA therapy compared to LAA (46%) and Crypt (42%) (*p* = 0.03). At admission, 59.3% of patients had severe stroke (NIHSS > 15), decreasing to 17.3% at discharge. NIHSS scores at discharge differed significantly (*p* = 0.04), with LAA patients more affected, as 28% had severe strokes (NIHSS > 15) compared to CE (16%) and Crypt (8%). Based on mRS scores at 90 days, 38.7% of patients had good outcomes (mRS 0–2), while 52.7% had poor outcomes (mRS 3–6). Successful mTICI 2c/3 recanalisation was achieved in 82.7% of patients, with the majority taking one pass (62.7%).

**Table 1.** Patient characteristics.

Clinical parameters	All patients (*n* = 150)	Etiology			χ^2^/H	*p*-Value
		LAA (*n* = 50)	CE (*n* = 50)	Crypt (*n* = 50)		
Sex						
Male, *n* (%)	81 (53.3)	35 (70)	23 (46)	23 (44)	7.1^a^	0.03*
Female, *n* (%)	67 (45.3)	15 (30)	26 (52)	26 (54)		
Age (year) (median, IQR)	71 (62, 79)	68 (62, 78)	74 (68, 82)	70 (55, 76)	4.2^b^	0.02^*^
Risk factors						
Hypertension, *n* (%)	100 (66.7)	37 (74)	34 (68)	29 (58)	2.9^a^	0.23
Hyperlipidemia, *n* (%)	68 (45.3)	23 (46)	26 (52)	19 (38)	0.4^a^	0.82
Atrial fibrillation, *n* (%)	42 (28)	6 (12)	32 (64)	4 (8)	48.4^a^	<0.001*
Diabetes mellitus, *n* (%)	22 (14.7)	5 (10)	15 (30)	2 (4)	14.8^a^	<0.001*
Smoking, *n* (%)	40 (26.7)	18 (36)	11 (22)	11 (22)	3.3^a^	0.19
Coronary artery diseases, *n* (%)	31 (20.7)	11 (22)	15 (30)	5 (10)	6.2^a^	0.04*
rtPA						
Yes, *n* (%)	54 (37)	23 (46)	11 (22)	20 (42)	6.8^a^	0.03^*^
No, *n* (%)	96 (63)	27 (54)	39 (78)	30 (58)		
NIHSS (admission)						
Mild to moderate (⩽15), *n* (%)	58 (38)	16 (32)	21 (42)	21 (40)	1.9^a^	0.38
Severe (>15), *n* (%)	88 (59.3)	34 (68)	28 (56)	26 (54)		
NIHSS (discharged)						
Mild to moderate (⩽15), *n* (%)	109 (72.7)	33 (66)	36 (72)	40 (80)	6.3^a^	0.04*
Severe (>15), *n* (%)	26 (17.3)	14 (28)	8 (16)	4 (8)		
mRS (90 days)						
0–2, *n* (%)	59 (38.7)	14 (28)	21 (42)	24 (46)	4.8^a^	0.09
3–6, *n* (%)	78 (52.7)	32 (64)	24 (48)	22 (46)		
Final mTICI scores						
2c/3, *n* (%)	124 (82.7)	39 (78)	44 (88)	41 (82)	2.0^a^	0.37
0/1/2a/2b, *n* (%)	24 (16)	11 (22)	6 (12)	7 (14)		
Number of passes						
1, *n* (%)	94 (62.7)	36 (72)	30 (60)	28 (56)	2.6^b^	0.27
2, *n* (%)	31 (20.7)	7 (14)	8 (16)	16 (32)		
3, *n* (%)	13 (8.7)	5 (10)	4 (8)	4 (8)		
4, *n* (%)	4 (2.7)	1 (2)	3 (6)	0 (0)		
⩾5, *n* (%)	8 (5.3)	1 (2)	5 (10)	2 (4)		

a Analysed with the Chi-square χ^2^ test.

b Analysed with the Kruskal–Wallis H test.

* *p* < 0.05.

### Histological components across different etiology groups

The histological components of the clots were evaluated using MSB staining, as shown in Supplemental Figure 1. Supplemental Table 1 presents the quantification results for each component across different etiological groups. Although not significantly different, the RBCs% was highest in LAA, while the WBCs% was significantly higher in CE (6.0%) compared to LAA (4.5%) and Crypt (4.3%) (*p* = 0.006). These results are consistent with our previous findings.^22,28^

### Heterogeneous CRP expression in clot samples

**Figure 1.**
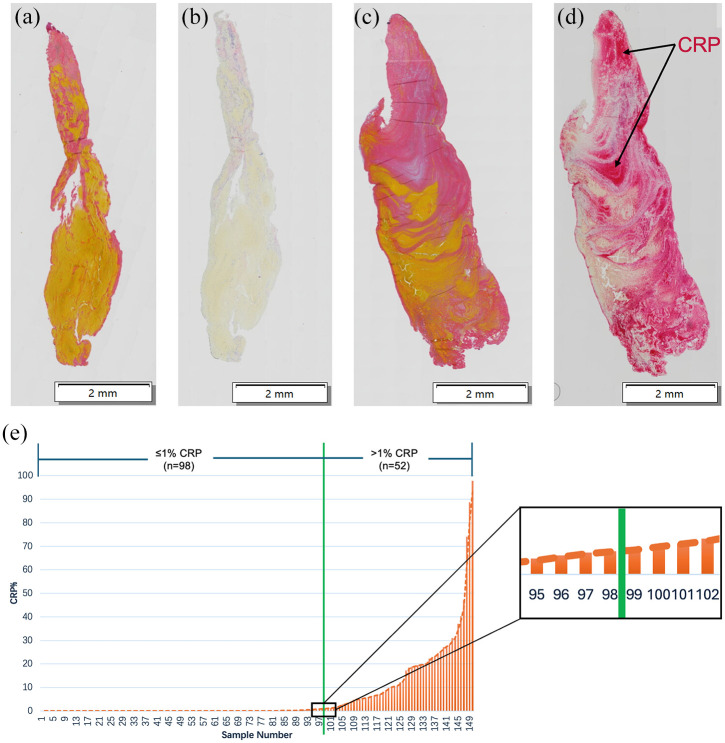
Heterogeneity in CRP expression across clot samples. Representative IHC staining images of samples with minimal CRP expression (b) and samples with substantial CRP expression (d), along with their corresponding MSB staining images (a and c). Scale bar: 2 mm. Distribution of samples ranked by CRP quantitative levels from low to high (e). The green line represents the 1% CRP expression cut-off threshold (between samples 98 and 99).

CRP expression in the clots was assessed using IHC staining. The results showed significant variability in CRP expression among the samples. Figure 1 presents representative IHC-stained images of CRP for two samples and their corresponding MSB staining images. One sample demonstrated minimal CRP (Figure 1(b)), while the other exhibited substantial CRP (Figure 1(d)).

Our observation that some samples showed high CRP levels while the majority had minimal levels led us to stratify them into two groups: CRP high and CRP low. To define the cut-off, we ranked the quantified data from low to high and found that approximately two-thirds of samples expressed minimal CRP, while one-third expressed substantial CRP (Figure 1(e)). The median value (0.04%) was too low to serve as an effective threshold cut-off point. The mean was also unsuitable due to the clearly skewed data distribution. Therefore, we determined that a value near the two-thirds mark may be a reasonable threshold value to explore the impact of heightened CRP on clots and patient characteristics. Of the 150 samples analysed, 98 had CRP levels below 1% (0%–0.96%, median: 0.0015%), while 52 had CRP levels above 1% (1.05%–97.85%, median: 11.45%) (Figure 1(e), Supplemental Table 2). LAA and Crypt showed more cases with minimal CRP (⩽1%) (70% and 74%), whereas CE had significantly more cases with substantial CRP (>1%) (48%; Table 2) (*p* = 0.048).

**Table 2.** CRP expression (minimal and substantial) by etiology.

CRP expression	All samples (*n* = 150)	Etiology		
		LAA (*n* = 50)	CE (*n* = 50)	Crypt (*n* = 50)
⩽1%, *n* (%)	98 (65)	35 (70)	26 (52)	37 (74)
>1%, *n* (%)	52 (35)	15 (30)	24 (48)	13 (26)
χ^2^		6.06		
*p*		0.048*		

Data were analysed using the Chi-square χ2 test.

* *p* < 0.05.

### Comparison of patient characteristics and histological components between minimal and substantial CRP expression groups

We compared patient characteristics and histological components between groups with minimal (⩽1%) and substantial (>1%) CRP to explore potential differences. Supplemental Table 3 indicated no significant differences in clinical parameters between these two groups. Regarding histological components, although not statistically significant, RBCs tended to be lower in samples with substantial CRP. In contrast, fibrin was significantly higher in samples with substantial CRP (28.9%) compared to those with minimal CRP (20.6%) (*p* = 0.005). No significant differences were observed for other components between the two groups, as detailed in Table 3.

**Table 3.** Comparison of histological components between samples with substantial (⩾1%) and minimal (<1%) CRP expression.

Components	All patients (*n* = 150)	CRP expression		*U*	*p*
		⩽1% (*n* = 98)	>1% (*n* = 52)		
RBCs%	44.8 (27.2, 56.5)	47.0 (30.4, 57.2)	40.0 (22.6, 55.3)	2162	0.13
WBCs%	4.7 (2.9, 7.5)	4.7 (2.7, 7.6)	5.0 (3.2, 7.4)	2340	0.41
Fibrin%	21.7 (13.0, 31.7)	20.6 (11.5, 28.4)	28.9 (15.5, 35.6)	1836	0.005*
Platelets%	24.5 (16.0, 37.6)	25.1 (16.0, 39.2)	23.7 (14.4, 35.8)	2310	0.35
Collagen%	0.01 (0.004, 0.03)	0.01 (0.004, 0.03)	0.01 (0.003, 0.03)	2504	0.86

Data were presented as median% (IQR) and analysed using the Mann–Whitney *U* test.

* *p* < 0.05.

To investigate whether clots containing substantial CRP were more commonly fibrin-high or WBC-high and whether this relationship varied by etiology, we classified all samples into fibrin-high and fibrin-low groups using the median fibrin level as the threshold. Independently, we also classified samples into WBC-high and WBC-low groups based on the median WBC level. Chi-square analysis showed that fibrin-high clots were more likely to contain substantial CRP in the CE (*p* = 0.049) and Crypt (*p* = 0.051) groups but not in the LAA group (*p* = 0.56). In contrast, there were no significant differences in the proportion of clots with substantial versus minimal CRP between the WBC-high and WBC-low groups (Supplemental Table 4).

Furthermore, we performed Spearman's correlation analysis to examine the relationship between CRP levels and various histological components. The results showed a significant but relatively weak positive correlation between CRP and fibrin (*r* = 0.28; *p* = 0.0006). Additionally, there was a weak inverse correlation between CRP and RBCs (*r* = −0.16; *p* = 0.052). No significant correlations were observed between CRP and WBCs, platelets, or collagen (Supplemental Figure 3).

### Co-localisation between CRP and main components

Following this, we conducted co-localisation studies to investigate the localisation of CRP within the clots. As seen in Supplemental Figure 2, CRP demonstrated strong co-localisation with fibrin (Fibrinogen, Row 1) in the clot samples, but not with platelets (CD42b, Row 2) and RBCs (Glycophorin A, Row 6). In addition, CRP exhibited partial co-localisation with neutrophils (CD66b, Row 3), macrophages (CD68, Row 4), and lymphocytes (CD3, Row 5). Notably, co-localisation appeared to be concentrated primarily on the cell surface of WBCs.

## Discussion

CRP is one of the most extensively studied inflammatory biomarkers, yet its presence in stroke thrombi has not been previously investigated. The presence of CRP in thrombi may indicate either systemic inflammation or a localised response to vascular injury. Elevated blood CRP levels, commonly associated with systemic inflammation,^29^ may become deposited in the thrombi, reflecting the body’s broader inflammatory state. Alternatively, studies suggest that CRP could be locally produced within the thrombus, particularly by monocytes and lymphocytes,^30^ indicating a localised inflammatory response. Given the pivotal role of inflammation in the pathogenesis of stroke,^31^ inflammatory biomarkers hold significant potential as etiological biomarkers for AIS.

In this study, we observed notable heterogeneity in CRP expression among clot samples, leading us to consider whether the presence of CRP in thrombi might indicate underlying factors. Given that our primary objective was to investigate whether CRP could serve as a potential etiological marker for AIS, we first examined whether CRP levels in different clot samples were related to etiology. Our findings revealed that more samples showed substantial CRP in CE-origin clots than those from LAA and Crypt, suggesting that inflammation may play an important role in the formation of clots, especially those of CE-origin. These results align with previous studies on CRP serum levels. Therefore, our findings further support the potential of CRP as an etiological biomarker for AIS, suggesting that AIS patients with elevated CRP levels in thrombus are more likely to have CE as the underlying etiology.

Next, we investigated the relationship between CRP and histological components. Our findings showed that samples with high CRP levels contained significantly more fibrin. Moreover, we observed a significant, but relatively weak, correlation between CRP and fibrin levels. No significant correlations were found between CRP and other histological components. These results suggest that AIS patients in an acute inflammatory state are more likely to develop fibrin-rich thrombi, which are harder to remove and often associated with worse outcomes.^32^ Therefore, assessing the inflammatory status of AIS patients may help predict clot types, enabling personalised treatment and prevention strategies.

To our knowledge, no prior investigations have examined the co-localisation of CRP and fibrinogen within thrombi. Our study demonstrated clear co-localisation between these two proteins, further supporting their strong association. Several factors may explain this relationship. On one hand, both CRP and fibrinogen are acute-phase inflammatory markers produced by the liver, and their levels may be simultaneously upregulated in patients with symptomatic coronary disease.^33^ This aligns with previous studies showing a positive correlation between CRP and fibrinogen levels in blood.^34,35^ On the other hand, earlier research suggests that CRP can bind to fibrinogen, potentially modifying fibrin structure.^36^ Furthermore, variations in CRP levels have also been shown to impact fibrin properties, such as clot permeability and lysability.^37^ Our findings extend the observed correlation between CRP and fibrinogen to blood clots, underscoring the role of inflammation in thrombosis. These results highlight CRP’s dual role as both an inflammation marker and an active participant in promoting thrombosis. Consequently, targeting inflammation could be crucial for preventing or managing thrombotic events.

Another question worth considering is the origin of CRP in the blood clots. While typically synthesised in the liver, recent studies suggest that endothelial cells, monocytes, and lymphocytes might also produce CRP locally.^38^ The co-localisation between CRP and fibrinogen indicates that CRP may deposit on thrombi by binding with fibrinogen. Additionally, CRP co-localised with WBCs, suggesting the potential for local synthesis by these cells. The co-localisation occurs predominantly at the surface of WBCs, indicating that it is more likely that circulating CRP is captured by combining with the receptors on their surface. Known CRP receptors include oxidised low-density lipoprotein (OxLDL), lectin-like oxidised LDL receptor-1 (LOX-1), complement protein C1q, class-A scavenger receptor (SR-A), and FCγ receptors (FcγRs).^38^ Among these, LOX-1, SR-A and FcγRs are present on the surface of WBCs.^39^ The binding of CRP to these receptors is associated with thrombus formation through endothelial^40^ and complement activation,^41,42^ contributing to a pro-thrombotic environment, particularly in cardiovascular diseases.^43^

Moreover, the co-localisation of CRP with fibrin and WBCs within thrombi could provide new insights into the etiological study of AIS. Previous studies have shown that fibrin-rich clots are more commonly associated with CE strokes, while RBC-rich clots are characteristic of LAA strokes. Additionally, WBCs are often more prominently expressed in clots from patients with CE strokes. Our chi-square analysis revealed that fibrin-high clots in the CE group were more likely to contain substantial CRP, whereas no such association was found in the LAA group. In contrast, the proportion of clots with substantial versus minimal CRP showed no significant differences between the WBC-high and WBC-low groups. This may be due to the low content of WBCs within thrombi and the limited sensitivity of the histological method. Given the sample size limitations, we must be cautious in drawing definitive conclusions. These results suggest that CRP may not be independently associated with CE etiology, as its relationship with fibrin cannot be overlooked. However, whether the high CRP expression observed in more CE clots is independently associated with CE etiology, or whether it is associated with higher fibrin or WBC content, remains unclear and warrants further investigation.

We also compared clinical parameters between thrombus samples with substantial or minimal CRP expression. Although no correlation was observed, possibly due to the limited sample size, previous studies have linked CRP to worse AIS outcomes. Targeting CRP with anti-inflammatory treatments may improve outcomes or prevent stroke recurrence, although the effectiveness of such treatments in reducing post-AIS risk remains unclear. Nevertheless, it appears that stroke etiology and inflammation state should be given key consideration.

This study has several limitations. Firstly, the absence of blood samples prevents the analysis of systemic CRP levels or WBC counts. Future investigations correlating CRP levels in thrombi with systemic CRP levels or WBC counts would provide valuable insights into the relationship between local and systemic inflammation. Secondly, due to the lack of reference points for CRP expression levels in clots, we defined 1% as the cut-off threshold for categorising CRP levels. This threshold may be a limitation, and future research should incorporate sensitivity analysis to refine it in conjunction with CRP blood levels in stroke patients. Thirdly, we did not explicitly exclude patients with inflammatory or autoimmune conditions that could elevate CRP levels. Addressing these potentially confounding factors in future studies would strengthen the findings. Finally, inter-centre variability may introduce bias. However, we minimised this risk by standardising clot collection and processing methods across stroke centres and conducting all clot analyses in a single-core laboratory to ensure consistency and reduce variability.

Our findings highlight the pivotal role of inflammation in AIS clot development and suggest the need for further research on inflammatory markers to refine etiological biomarkers, which is essential for optimising individual therapy for AIS patients. These findings could be instructive for selecting appropriate patients for anti-inflammatory therapy in future RCTs. Overall, this research could deepen our understanding of thrombus formation, leading to improved treatments, better patient prognosis, and enhanced secondary prevention strategies.

## Conclusion

Our findings show that high CRP levels are more common in AIS clots of CE etiology. Additionally, the strong link between CRP and fibrin within the thrombi is an interesting observation worthy of further study. Taken together, this study indicates that inflammation may play an important role in the formation of blood clots, especially fibrin-rich clots and those of CE-origin.

## Supplemental Material

sj-docx-1-eso-10.1177_23969873251315636 – Supplemental material for C-reactive protein expression in acute ischemic stroke blood clots: Implications for etiology Supplemental material, sj-docx-1-eso-10.1177_23969873251315636 for C-reactive protein expression in acute ischemic stroke blood clots: Implications for etiology by Wenyi Liu, Cansu Sahin, Nazan Güner Sak, Alice Giraud, Pierluca Messina, Franz Bozsak, Jean Darcourt, Federico Sacchetti, Anne-Christine Januel, Guillaume Bellanger, Jorge Pagola, Jesus Juega, Hirotoshi Imamura, Tsuyoshi Ohta, Laurent Spelle, Vanessa Chalumeau, Uros Mircic, Predrag Stanarčević, Ivan Vukašinović, Marc Ribo, Nobuyuki Sakai, Christophe Cognard and Karen Doyle in European Stroke Journal
