# Supplementary material for: C-reactive protein expression in acute ischemic stroke blood clots: Implications for etiology
Source: Eur Stroke J. 2025 Feb 5;10(3):892–901. doi: 10.1177/23969873251315636 (PMC11803589; doi:10.1177/23969873251315636)
Supplement: sj-docx-1-eso-10.1177_23969873251315636 – Supplemental material for C-reactive protein expression in acute ischemic stroke blood clots: Implications for etiology [file sj-docx-1-eso-10.1177_23969873251315636.docx]

**Supplemental materials**


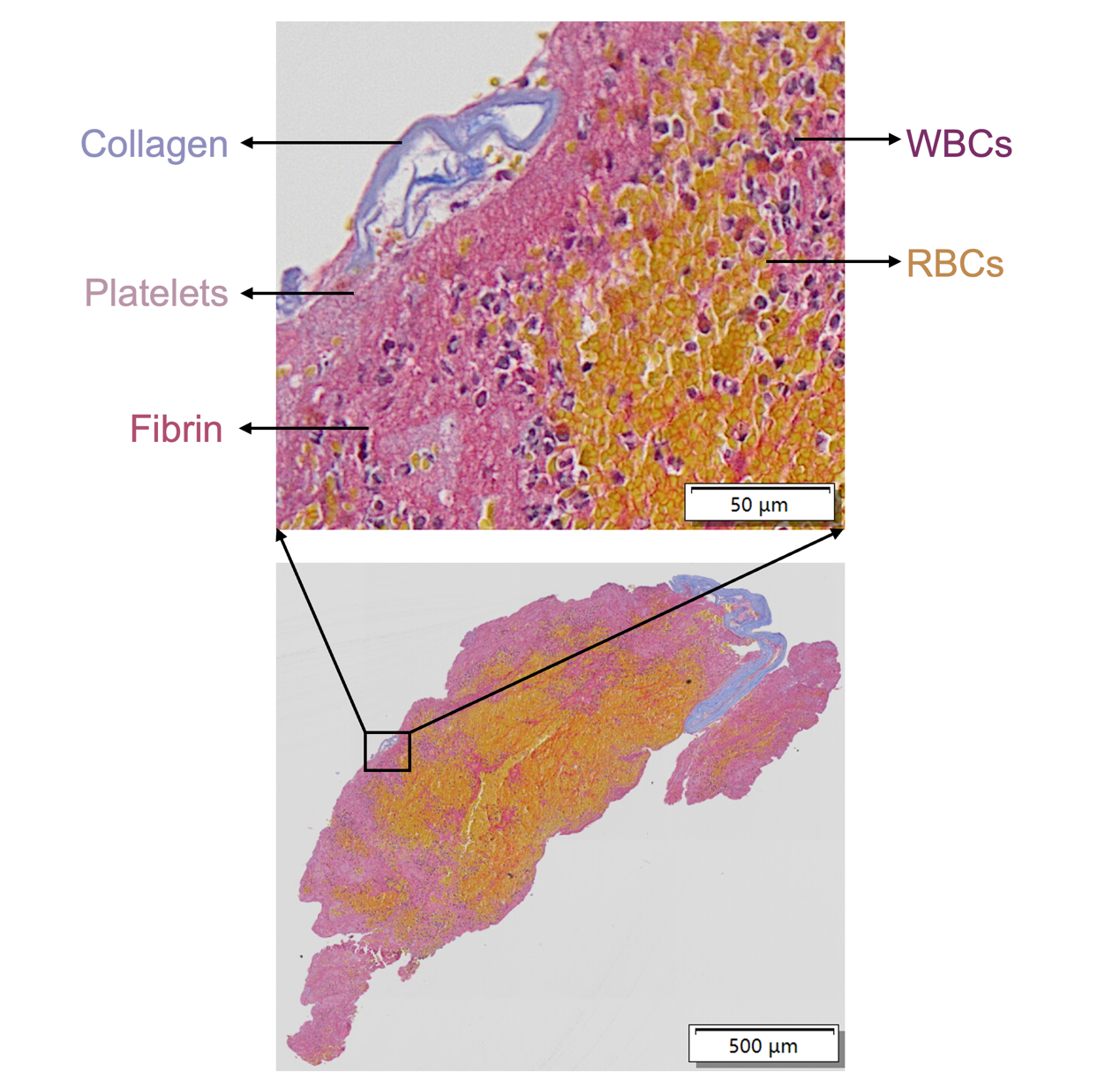


**Supplemental Figure 1.** Representative MSB staining images showing RBCs (yellow), WBCs (purple), fibrin (red), platelets (grey or pinkish), and collagen (blue). Scale bar: 50μm and 500 μm.


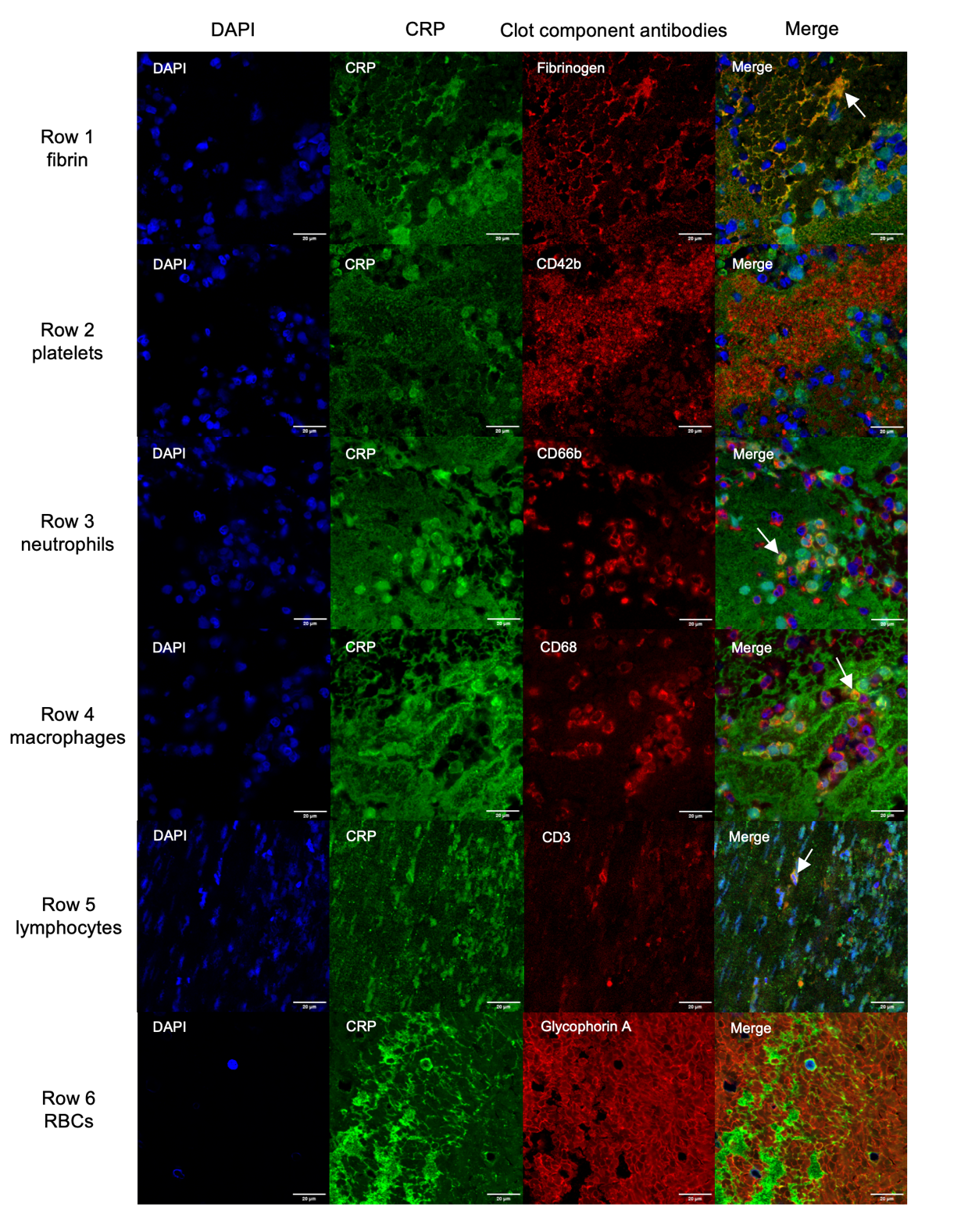


**Supplemental Figure 2.** Representative immunostaining images for blood clots (CRP, green; Fibrinogen, CD42b (platelets marker), CD66b (neutrophils marker), CD68 (macrophages marker), CD3 (lymphocytes marker), Glycophorin A (RBCs marker) red). Scale bar: 20μm.

**Supplemental Table 1.** Histological components of blood clots from different etiological groups.

|  |  | Etiology | | |  | |  |
| --- | --- | --- | --- | --- | --- | --- | --- |
| Components | All samples (n=150) | LAA (n=50) | CE (n=50) | Crypt (n=50) | H | *p* | |
| RBCs% | 44.8 (27.2, 56.5) | 47.5  (31.8, 57.5) | 44.3 (28.6, 57.2) | 42.0 (25.2, 54.4) | 0.8 | 0.67 | |
| WBCs% | 4.7 (2.9, 7.5) | 4.5 (2.5, 7.3) | 6.0 (4.1, 10.9) | 4.3 (2.7, 6.4) | 10.2 | **0.006*** | |
| Fibrin% | 21.7 (13.0, 31.7) | 24.0 (13.4, 32.2) | 21.2 (14.8, 31.7) | 21.4 (8.3, 31.7) | 1.1 | 0.58 | |
| Platelets% | 24.5 (16.0, 37.6) | 25.9 (12.9, 36.3) | 22.8 (16.6, 33.8) | 26.9 (20.0, 41.9) | 3.6 | 0.17 | |
| Collagen% | 0.01 (0.004, 0.03) | 0.01 (0.002, 0.05) | 0.01 (0.003, 0.02) | 0.01 (0.005, 0.04) | 1.7 | 0.43 | |
| *Note:* Data were presented as median (IQR) and analysed using the Kruskal-Wallis test. **p*<0.05 | | | | | | | |

**Supplemental Table 2.** The descriptive statistics of all clot samples and subgroups with minimal (≤1%) and substantial (>1%) CRP expression.

|  |  | CRP expression | |
| --- | --- | --- | --- |
| Descriptive statistical parameters | All samples (n=150) | ≤1%  (n=98) | >1% (n=52) |
| Median% | 0.04 | 0.0015 | 11.45 |
| IQR (Q1%, Q3%) | (0.0008, 5.60) | (0.0003, 0.04) | (5.11, 24.41) |
| Minimum% | 0 | 0 | 1.05 |
| Maximum% | 97.85 | 0.96 | 97.85 |
| Mean% | 6.49 | 0.08 | 18.57 |
| Std. Deviation | 14.9 | 0.19 | 20.52 |
| Std. Error of Mean | 1.22 | 0.02 | 2.84 |

**Supplemental Table 3.** Comparison of patient characteristics between samples with substantial (>1%) and minimal (≤1%) CRP expression.

|  |  | CRP expression | |  |  |
| --- | --- | --- | --- | --- | --- |
| Clinical parameters | All patients (n=150) | ≤1% (n=98) | >1% (n=52) | X^2^/U | *p* value |
| Sex |  |  |  |  |  |
| Male, n (%) | 81 (54) | 53 (54.1) | 28 (53.8) | 0.02^a^ | 0.87 |
| Female, n (%) | 67 (44.7) | 43 (43.9) | 24 (46.2) |  |  |
| Age (year) (median, IQR) | 71 (62, 79) | 71 (61, 79) | 72 (66, 80) | 2339^b^ | 0.41 |
| Risk factors |  |  |  |  |  |
| hypertension, n (%) | 100 (66.7) | 65 (66.3) | 35 (67.3) | 0.01^a^ | 0.90 |
| hyperlipidemia, n (%) | 68 (45.3) | 45 (45.9) | 23 (44.2) | 0.04^a^ | 0.84 |
| atrial fibrillation, n (%) | 42 (28.0) | 24 (24.5) | 18 (34.6) | 1.7^a^ | 0.19 |
| diabetes mellitus, n (%) | 22 (14.7) | 13 (13.3) | 9 (17.3) | 0.4^a^ | 0.50 |
| smoking, n (%) | 40 (26.7) | 26 (26.5) | 14 (26.9) | 0.003^a^ | 0.96 |
| coronary artery diseases, n (%) | 31 (20.7) | 16 (16.3) | 15 (28.8) | 3.2^a^ | 0.07 |
| rtPA |  |  |  |  |  |
| Yes, n (%) | 54 (36) | 39 (39.8) | 15 (28.8) | 1.8^a^ | 0.18 |
| No, n (%) | 96 (64) | 59 (60.2) | 37 (71.2) |  |  |
| NIHSS (admission) |  |  |  |  |  |
| Mild to moderate (≤15), n (%) | 58 (38.7) | 37 (37.8) | 21 (40.4) | 0.01^a^ | 0.90 |
| Severe (>15), n (%) | 88 (58.7) | 57 (58.2) | 31 (59.6) |  |  |
| NIHSS (discharged) |  |  |  |  |  |
| Mild to moderate (≤15), n (%) | 109 (72.7) | 72 (73.5) | 37 (71.2) | 0.04^a^ | 0.84 |
| Severe (>15), n (%) | 26 (17.3) | 16 (16.3) | 9 (17.3) |  |  |
| mRS (90 days) |  |  |  |  |  |
| 0-2, n (%) | 59 (39.3) | 39 (39.8) | 20 (38.5) | 0.15^a^ | 0.70 |
| 3-6, n (%) | 78 (52.0) | 57 (58.2) | 29 (55.8) |  |  |
| final mTICI scores |  |  |  |  |  |
| 2c/3, n (%) | 124 (82.7) | 80 (81.7) | 44 (84.6) | 0.04^a^ | 0.84 |
| 0/1/2a/2b, n (%) | 24 (16) | 16 (16.3) | 8 (15.4) |  |  |
| number of passes |  |  |  |  |  |
| 1, n (%) | 94 (62.7) | 61 (62.2) | 33 (63.5) | 4.1^a^ | 0.40 |
| 2, n (%) | 31 (20.7) | 23 (23.5) | 8 (15.4) |  |  |
| 3, n (%) | 13 (8.7) | 8 (8.2) | 5 (9.6) |  |  |
| 4, n (%) | 4 (2.7) | 1 (1) | 3 (5.8) |  |  |
| ≥ 5, n (%) | 8 (5.3) | 5 (5.1) | 3 (5.8) |  |  |
| *Note*: ^a^Analysed with the Mann-Whitney U test; ^b^Analysed with the Chi-square X^2^ test. | | | | | |

**Supplemental Table 4.** Comparison the proportion of CRP substantial (>1%) and minimal (≤1%) expressed clots across fibrin-high/low and WBCs-high/low clot samples.

|  | All, n=150 | | LAA, n=50 | | CE, n=50 | | Crypt, n=50 | | |
| --- | --- | --- | --- | --- | --- | --- | --- | --- | --- |
|  | ≤1% CRP, n | >1% CRP, n | ≤1% CRP, n | >1% CRP, n | ≤1% CRP, n | >1% CRP, n | ≤1% CRP, n | >1% CRP, n | |
| Fibrin-high | 43 | 32 | 20 | 8 | 9 | 15 | 14 | 9 | |
| Fibrin-low | 54 | 21 | 14 | 8 | 17 | 9 | 23 | 4 | |
| X^2^ | 3.5 | | 0.34 | | 3.89 | | 3.82 | | |
| *p* | 0.06 | | 0.56 | | **0.049*** | | 0.051 | | |
| WBC-high | 47 | 28 | 15 | 7 | 19 | 14 | 13 | 7 | |
| WBC-low | 51 | 24 | 20 | 8 | 7 | 10 | 24 | 6 | |
| X^2^ | 0.47 | | 0.06 | | 1.21 | | 1.40 | | |
| *p* | 0.49 | | 0.80 | | 0.27 | | 0.24 | | |
| *Note*: Data were analysed using the Chi-square test. **p*<0.05 | | | | | | | | |  |


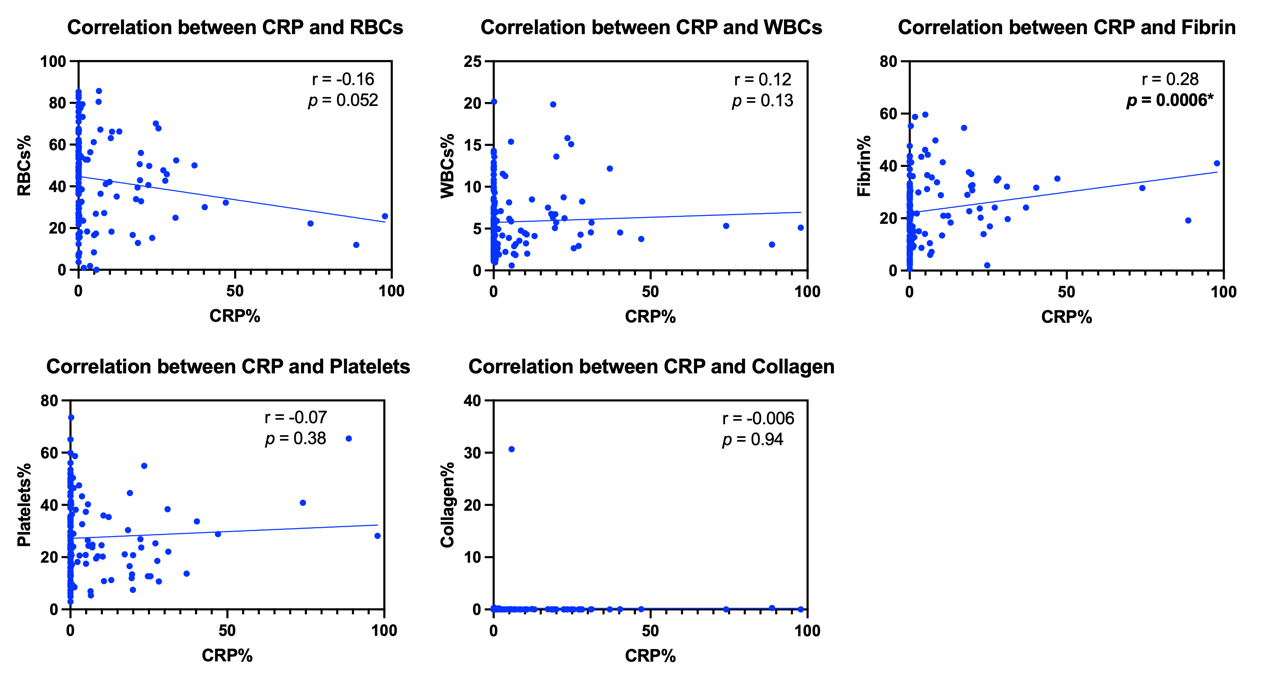


**Supplemental Figure 3.** The correlation between CRP and the main histological components within blood clots. Spearman’s correlation analysis was used. **p*<0.05.
